# Greater Similarity Between L1 and L2’s Brain Network in Adults Than in Children

**DOI:** 10.3389/fnins.2022.816729

**Published:** 2022-04-25

**Authors:** Fan Cao, Yuyu Fan, Xin Yan, Wuying Chen, Maddie Dodson-Garrett, Gregory J. Spray, Zhao Wang, Yuan Deng

**Affiliations:** ^1^Department of Psychology, Sun Yat-sen University, Guangzhou, China; ^2^Department of Communicative Sciences and Disorders, Michigan State University, East Lansing, MI, United States; ^3^School of Education Science, Jiangsu Normal University, Xuzhou, China; ^4^School of Chinese Language and Literature, Shaanxi Normal University, Xi’an, China; ^5^CAS Key Laboratory of Behavioral Science, Institute of Psychology, Chinese Academy of Sciences, Beijing, China

**Keywords:** bilingual, fMRI, development, rhyming, Chinese-English

## Abstract

It has been documented that processing L2 and L1 engages a very similar brain network in bilingual adults. However, it is not known whether this similarity is evident in bilingual children as well or it develops with learning from children to adults. In the current study, we compared brain activation in Chinese-English bilingual children and adults during L1 and L2 processing. We found greater similarity between L1 and L2 in adults than in children, supporting the convergence hypothesis which argues that when the proficiency of L2 increases, the L2’s brain network converges to the L1’s brain network. We also found greater differences between adults and children in the brain for L2 processing than L1 processing, even though there were comparable increase in proficiency from children to adults in L1 and L2. It suggests an elongated developmental course for L2. This study provides important insights about developmental changes in the bilingual brain.

## Introduction

There has been a consensus among researchers that brain networks involved in L1 and L2 processing are mostly overlapping in bilingual adults with subtle accommodations to the special features of the language (Perani et al., 1998; Chee et al., 1999a; Klein et al., 1999; Chee et al., 2000; Tettamanti et al., 2002; Musso et al., 2003; Sakai et al., 2004; Perani and Abutalebi, 2005). This neuroimaging finding of great overlap between L1 and L2 is consistent with the repeated observation from behavioral studies that L1 influences the organization of L2’s representations (Wu and Thierry, 2010; Costa et al., 2016), suggesting an extensive transfer from L1 to L2 (Koda, 1990; Kroll et al., 2012). The finding of similar brain activation in L1 and L2 also lines up with the unified model proposed based on computational modeling studies, suggesting that the same computational principles can be applied in L1 and L2 language acquisition (MacWhinney, 2012). A recent study further suggests that higher L2 proficiency is related to greater similarity between L1 and L2 in brain activation in Chinese-English late bilingual adults (Cao et al., 2013a). Similarly, another study found that higher proficiency is associated with greater overlap between L1 and L2 in Italian-English bilinguals (Perani et al., 1998). The proficiency effect found in the previous two studies is supportive of the convergence hypothesis proposed by Green (2003), which argues that differences between native speakers and L2 speakers disappear as L2 proficiency increases. This hypothesis has so far been supported by a number of previous studies with a manipulation of L2 proficiency (Chee et al., 1999b; De Bleser et al., 2003; Briellmann et al., 2004; Stein et al., 2009). Taken together, previous studies suggest an overlapped network in L1 and L2, with L2 proficiency being an important variable influencing the degree of similarity (Hernandez et al., 2007).

How L1 and L2 are processed similarly or differently in bilingual children has been studied much less than in bilingual adults. A handful of studies have been published until today, and they suggest an overlapped network between L1 and L2, such as in a semantic judgment task in late Chinese-English bilingual children (Xue et al., 2004), and in a word reading task in simultaneous Hindi-English bilingual children (Cherodath and Singh, 2015). These studies also suggest some activation differences that may be due to different proficiency level. For example, a functional near-infrared spectroscopy (fNIRS) study found greater activation for L1 than L2 in a word repetition task at the bilateral middle/superior temporal gyrus, angular gyrus, supramarginal gyrus, and inferior frontal gyrus (IFG) in 6–10 years old Japanese-English bilingual children who learn English as a foreign language (Sugiura et al., 2011), which may be driven by greater proficiency in L1 than L2. Another study found English-Chinese bilingual children showed similarities between L1 and L2 in an auditory morphological task while the left IFG was more activated for English than Chinese which may be due to the higher proficiency in English than Chinese as well (Ip et al., 2016). In summary, according to the current literature, bilingual children also show an overlap between L1 and L2 brain networks.

However, what is lacking in the literature is the understanding of the dynamic change between L1 and L2 over development. For example, we do not know whether the brain network for L1 and L2 becomes more convergent or more differentiated with learning and development. According to the convergence hypothesis by Green (2003), one would expect greater convergence between L1 and L2 in adults than in children because the differences between the first and second language disappear as L2 proficiency increases. However, according to the interactive specialization model (Johnson, 2011), a specific brain region becomes specialized to a certain type of stimuli or calculations with learning and development. One would expect greater differentiation between L1 and L2 in adults than in children due to increased specialization to each language. Until today, there has been only one study that directly examined the developmental changes of brain activations in both L1 and L2 in bilinguals (Hernandez et al., 2015).

In Hernandez et al.’s (2015) study, it was found that during a single word reading task, Spanish-English bilingual adults showed greater activation in the bilateral middle temporal gyrus in reading English than bilingual children, while only the right middle temporal gyrus was more activated in adults than children in reading Spanish, suggesting greater developmental changes for L2, English than for L1, Spanish. This was explained by the fact that adults had a higher English proficiency than children, whereas their proficiency was comparable to children in Spanish. In other words, this study captured the transition from Spanish dominance in children to English dominance in adults. Therefore, the greater developmental change for English than Spanish in the brain is simply driven by greater proficiency change in English than in Spanish in this study. An ideal situation to study whether L1 and L2 are more convergent or differentiated in the brain over development is to have a bilingual population who have similar amount of growth in L1 and L2 across development, so that the increased/decreased language difference in the brain cannot be driven by unequal proficiency change in the two languages over development.

In the current study, we directly compared brain activation of L1 and L2 in Chinese-English bilingual children and adults who showed comparable increase in the proficiency of Chinese and English over development. We expected to observe greater similarity between L1 and L2 in brain activation in adults than in children if there is increased language convergence as proficiency increases. We expected less similarity between L1 and L2 in adults than in children, if there is increased differentiation between L1 and L2 as proficiency increases.

## Materials and Methods

### Participants

Two groups of native Chinese adults and two groups of native Chinese children were recruited in Beijing, China. Adult participants were undergraduate or graduate students at Beijing Normal University, who were randomly assigned to perform a Chinese word rhyming judgment task [i.e., the adult Chinese (AC) group] (*N* = 20, mean age = 21 years), or to perform an English pseudoword rhyming judgment task [i.e., the adult English (AE) group] (*N* = 15, mean age = 22.9 years). The Chinese children were fifth-graders recruited from eight public elementary schools in Beijing. One group of children performed the Chinese word rhyming judgment task [i.e., the child Chinese (CC) group] (*N* = 14, mean age = 11 years) and the other group performed the English pseudoword rhyming judgment task [i.e., the child English (CE) group] (*N* = 13, mean age = 11 years). CE and CC were matched on age [*t*(25) = 0.528, *p* > 0.05], performance on a Chinese character naming test [*t*(25) = 0.018, *p* > 0.05] and a Chinese reading fluency test [*t*(25) = 0.225, *p* > 0.05] (Table 1).

**TABLE 1:** Demographic information and testing scores for the four groups of participants.

Mean (standard deviation)	AC	AE	CC	CE
*N*	20	15	14	13
Age	21.45 (2.24) years	22.86 (2.26) years	134.38 (6.05) months	138.71 (5.54) months
AOA (years)		12.25 (0.62)		7.50 (2.10)
Chinese character naming (raw score)	–	–	124.00 (7.49)/150	124.08 (14.20)/150
Chinese reading fluency (raw score)	–	–	54.79 (13.49)/100	56.54 (14.54)/100
English synonym (raw score)	–	11.00 (5.48)/29	–	2.33 (2.19)/29
English Antonym (raw score)	–	15.13 (5.04)/29	–	4.92 (3.32)/29
Word ID (raw score)	–	43.27 (8.56)/60	–	20.00 (9.42)/60
Word Attack (raw score)	–	21.13 (5.51)/31	–	11.38 (7.56)/31
English reading fluency (raw score)	–	46.60 (13.21)/98	–	21.54 (9.02)/98
Rhyming judgment accuracy	0.93 (0.05)	0.82 (0.06)	0.82 (0.13)	0.67 (0.07)
Rhyming judgment reaction time	1224 (328)	1292 (365)	1655 (293)	1372 (375)

*AC, adults doing the Chinese task; AE, adults doing the English task; CC, children doing the Chinese task; CE, children doing the English task.*

According to the definition of late bilinguals by previous studies (Wartenburger et al., 2003; Berken et al., 2015), which is people who started to learn a second language at age 5 or later, all participants in our study were late Chinese-English bilinguals. The English proficiency of AE and CE was assessed with a standardized test, Woodcock Johnson-III (Woodcock et al., 2001) that includes five subtests of Antonyms, Synonyms, Word Identification, Word Attack, and Reading Fluency. Both Antonym and Synonym tested participants’ vocabulary; Word Identification tested word reading accuracy; Word Attack tested pseudoword reading accuracy; Reading Fluency tested reading speed and comprehension. AE was significantly higher than CE on all five English tests [*t*(25) = 6.041, *p* < 0.001 for antonym; *t*(25) = 5.146, *p* < 0.001 for synonym; *t*(26) = 6.850, *p* < 0.001 for Word ID; *t*(26) = 3.936, *p* < 0.01 for Word Attack; *t*(25) = 5.766, *p* < 0.001 for Reading Fluency] (Table 1). Participants in both AE and CE had not lived in an English immersion environment, and they do not use English in everyday life outside the classroom. Therefore, the immersion level and usage of English is comparable in AE and CE.

All participants were right-handed, free of any neurological disease or psychiatric disorders, did not have attention deficit hyperactivity disorder, and did not have any learning disabilities. The Institutional Review Board at Beijing Normal University and Michigan State University approved the consent procedures of the proposed study.

### Stimuli and Tasks

Participants performed a rhyming judgment task during magnetic resonance imaging (MRI) scanning. For the Chinese word rhyming judgment task, Chinese words consisted of two characters without homophones at the word level. There were 24 trials in each of 4 conditions, 2 rhyming and 2 non-rhyming. The 4 conditions were: (1) similar orthography and phonology (O+P+; e.g., 弥补/mi2bu3/, 纯朴/chun2pu3/), (2) similar orthography and different phonology (O+P-; e.g., 翻译/fan1yi4/, 选择/xuan3ze2/), (3) different orthography and similar phonology (O-P+; e.g., 环保/huan2bao3/, 大炮/da4pao4/), and (4) different orthography and phonology (O-P-; e.g., 损坏/sun3huai4/, 学科/xue2ke1/). Rhyming was defined as the same rime for the second character in each word pair. Orthographically similar words were defined as sharing the same phonetic radical for the second character of the word pair. Participants were told to ignore the tone when making the Chinese rhyming judgment. However, in order to avoid the influence of tonal information on the rhyming judgment, in half of the trials, the second character of the first and second word had the same tone (e.g., 弥补/mi2bu3/, 纯朴/chun2pu3/), and in the other half, they had different tones (e.g., 逮捕/dai4bu3/, 胸脯/xiong1pu2/). The word stimuli and the second character in each word were matched on adult written frequency (Beijing Language and Culture University, 1990), and number of strokes across conditions.

For the English pseudoword rhyming judgment task, all pseudoword stimuli were monosyllabic and there were 2 rhyming conditions: (1) similar orthographic and phonological endings (O+P+; e.g., *weat-yeat*), (2), different orthographic but similar phonological endings (O-P+; e.g., *nean-leen*) and equal number of non-rhyming trials with different orthographic and phonological endings (O-P-; e.g., *wub-hafe*). There were 24 trials in each of the 2 rhyming conditions and 48 trials in the non-rhyming condition. Only the rhyming trials were included in data analysis for both the Chinese word rhyming judgment task and the English pseudoword rhyming judgment task, because a non-rhyming judgment could be made based on the whole syllable without the need to segment the syllable to rime and onset and then compare the rime. Pseudowords were used in the English task, because we wanted to avoid the possibility of different levels of familiarity to English words in children and adults.

### Procedures

A pair of stimuli (either English pseudowords for the AE and CE groups, or Chinese words for the AC and CC groups) were sequentially presented in the visual modality and participants were asked to respond whether the two stimuli rhymed or not as quickly and as accurately as possible, using their right index finger for “yes” and their right middle finger for “no.” Each stimulus was presented for 800 ms, with a 200 ms interval between stimuli. A red fixation cross appeared on the screen immediately after the offset of the second word/pseudoword in the stimuli pair, indicating the need to respond. The response interval duration was variable (2200, 2600, or 2800 ms), such that each trial lasted for either 4000, 4400, or 4800 ms. Perceptual trials (24) were also included in which it required participants to determine whether two sequentially presented visual symbol patterns were matched or mismatched by pressing the “yes” or “no” buttons. There were also 48 null trials included as a baseline, in which it required participants to press the “yes” button when a fixation cross at the center of the screen turned from black to red. The timing for the perceptual and null trials was the same as the lexical trials.

### Magnetic Resonance Imaging Data Acquisition

All images were acquired using a 3.0 Tesla Siemens scanner (Siemens Healthcare, Erlangen, Germany) at Beijing Normal University where participants lay in the MRI scanner with their head position secured with foam padding. An optical response box was placed in each participant’s right hand and a compression alarm ball in the left hand. The head coil was positioned over each participant’s head in a way that they could effectively use a mirror to view the projection screen at the rear of the scanner. Gradient echo localizer images were acquired to determine the placement of the functional slices. For the functional images, a susceptibility weighted single-shot echo planar imaging (EPI) method with blood oxygenation level-dependency (BOLD) was used with the following scan parameters: time echo (TE) = 20ms, flip angle = 80°, matrix size = 128 × 128, field of view = 220 × 220 mm, slice thickness = 3 mm (0.48 gap), number of slices = 33, time repetition (TR) = 2,000 ms. These parameters resulted in a 1.7 × 1.7 × 3 mm voxel size. 145 whole-brain volumes were acquired each run with an interleaved bottom to top sequence, with one complete volume collected every 2 s. A high resolution, T1 weighted 3D image was also acquired with the following parameters: TR = 2300 ms, TE = 3.36 ms, flip angle = 9°, matrix size = 256 × 256, field of view = 256 mm, slice thickness = 1 mm, number of slices = 160, resulting voxel size = 1 × 1 × 1 mm. The acquisition of the anatomical scan took approximately 9 min.

### Image Analysis

Data analysis was performed using Statistical Parameter Mapping (SPM12).^1^ The following steps were used for data preprocessing. (1) Slice timing correction for interleaved acquisition using sinc interpolation. (2) 4th degree b-splice interpolation for realignment to the first volume. (3) Trilinear coregistration with the anatomical image. (4) Segmentation of the anatomical image. (5) Normalization of all functional brains to the Montreal Neurological Institute (MNI) transforms derived from the segmentation of the structural image. (6) 4 × 4 × 8 mm full width half maximum Gaussian kernel smoothing.

Statistical analyses at the first level were calculated using an event-related design with all lexical conditions, the perceptual control condition, and the null baseline condition. A high pass filter with a cutoff period of 128 s was applied. Trials were modeled using a canonical hemodynamic response function (HRF). Data from each subject were entered into a general linear model using an event-related analysis procedure. Group results were obtained using random-effects analysis by combining subject-specific summary statistics across the group as implemented in SPM12. The contrast of rhyming trials (including O+P+ and O-P+) versus the perceptual trials was entered into a flexible factorial design: an age (adults, children) by language (Chinese, English) ANCOVA with task accuracy as a covariate. Age effect, language effect and the interaction between them were calculated and reported. All reported results were at uncorrected *p* < 0.001 at the voxel level, and FDR corrected *p* < 0.05 at the cluster level.

In order to examine the similarity between brain activation of L1 and L2 within each age group, we calculated the similarity between CE and CC, and between AE and AC using the Dice Similarity Coefficient (DSC) formula (Dice, 1945; Craddock et al., 2012): 2| A ∩ B|/| A| + |B|, where A ∩ B is the number of voxels activated in the overlap between A and B, and | A| + |B| is the sum of brain voxels that were activated in A and B. The DSC ranges between 0 (no similarity) to 1 (perfect similarity). Therefore, the DSC is expected to inform the degree of similarity between A and B.

In order to examine what drove the interaction of age and language, we ran separate contrasts to examine developmental changes within each language (i.e., English: adults > children, and children > adults; Chinese: adults > children, and children > adults). We also broke down the interaction by looking at language Differences within each age group (i.e., adults: Chinese > English, and English > Chinese; children: Chinese > English, and English > Chinese). These contrasts were calculated with accuracy on the task regressed out. Then by identifying the contrasts that show common effect as the interaction, we can explain what drove the interaction.

For regions that showed a significant interaction in the last step, we also examined brain-behavioral correlation to examine whether these regions are also associated with English proficiency separately in AE and CE. The averaged accuracy on Synonym, Antonym, Word Attack, Word ID, and Reading Fluency subtests represented English proficiency.

## Results

### Behavioral Results

We conducted an age (children, adults) by language (Chinese, English) ANOVA on accuracy and reaction time of the rhyming judgment task. We found a significant main effect of age with adults being more accurate [*F*(1,58) = 47.289, *p* < 0.001] and faster [*F*(1,58) = 8.512, *p* < 0.01] than children. We found a significant main effect of language with Chinese being more accurate [*F*(1,58) = 67.370, *p* < 0.001] than English. The main effect of language was not significant for reaction time [*F*(1,58) = 1.518, *p* > 0.05]. The interaction was not significant for either accuracy [*F*(1,58) = 3.253, *p* > 0.05] or reaction time [*F*(1,58) = 4.009, *p* > 0.05] (Table 1).

### Brain Activation Results

#### The Main Effect of Language

**FIGURE 1:**
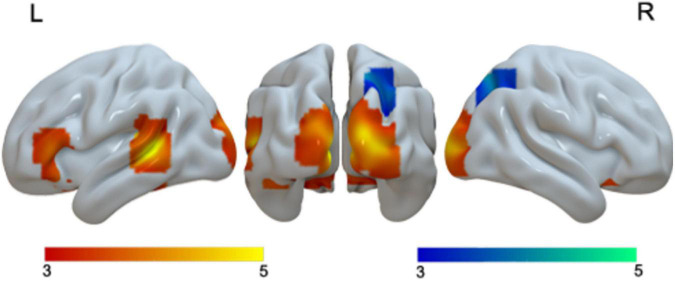
Brain activation in the main effect of language. Brain regions that were more activated in Chinese than in English are in red; brain regions that were more activated in English than in Chinese are in blue.

We found greater activation for Chinese than English at the left STG, bilateral lingual gyri and cuneus, bilateral putamen, and the left IFG. We found greater activation for English than Chinese in the right superior parietal lobule (SPL) (Table 2 and Figure 1).

**TABLE 2:** Brain regions that showed a significant main effect of language, a significant main effect of age, and a significant interaction effect between language and age.

Anatomical Region	H	BA	Voxels	x	y	z	Z

Main effect of language*: Chinese* > *English*							
Superior temporal gyrus	L	22	126	−51	−40	8	5.93
Lingual gyrus, cuneus	L, R	17, 18, 19	1217	−6	−70	−4	5.77
Putamen	L		168	−18	11	−4	5.31
Putamen	R		117	9	8	−1	4.31
Inferior frontal gyrus	L	45	35	−48	29	8	3.99

**Main effect of language*: English* > *Chinese***							

Superior parietal lobule	R	7	44	24	−67	44	4.26

**Main effect of age: *Adults* > *children***							

−							

**Main effect of age: *Children* > *Adults***							

Fusiform gyrus	R	37	106	30	−52	−13	4.64

**Interaction effect**							

Inferior frontal gyrus, Middle frontal gyrus,	R	9	31	54	17	38	4.62
Post-central gyrus	R	2	25	45	−31	47	4.42
Medial frontal gyrus	L/R		52	−12	38	−7	4.07

*H, hemisphere; L, left; R, right; BA, Brodmann area; Voxels, the number of voxels in each cluster; x,y,z, coordinates in the MNI atlas; Z: z-value of the peak voxel in the contrast.*

**FIGURE 2:**
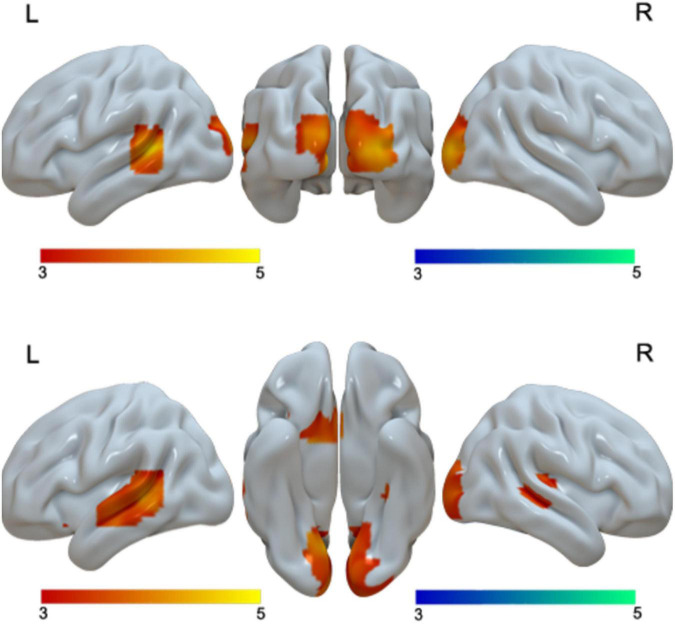
First row, brain regions that showed greater activation in Chinese than in English for adults. Second row, brain regions that showed greater activation in Chinese than in English for children. No brain regions showed greater activation in English than in Chinese for either adults or children.

When we examined language differences separately for adults and children, we found greater activation in Chinese than in English in the bilateral cuneus and left STG in adults, and greater activation in bilateral lingual/MOG, bilateral STG/MTG and left caudate in Chinese than in English for children. Neither children nor adults showed greater activation in English than in Chinese at the current threshold (Table 3 and Figure 2). However, when we lowered the threshold to *p* < 0.005 uncorrected, Cluster = FDR corrected *P* < 0.05, children showed greater activation in English than in Chinese in the right SPL. The DSC between Chinese and English is 0.26 for adults and 0 for children at the threshold of *p* < 0.001 uncorrected voxel level and 0 at the cluster level.

**TABLE 3:** Brain regions that showed significant differences between Chinese and English in either adults or children.

Anatomical region	H	BA	Voxels	x	y	z	Z

Adults*: Chinese* > *English*							
Cuneus	L/R		527	15	−91	8	5.02
Superior temporal gyrus	L	22	36	−51	−40	8	5.01

**Adults*: English* > *Chinese***							

–							

**Children*: Chinese* > *English***							

Superior temporal gyrus	R	22	38	39	−28	−7	4.94
Middle temporal gyrus	L	22	91	−51	−37	2	4.93
Lingual gyrus	L	18	338	−6	−67	−1	4.84
Caudate	L		126	−15	11	−1	4.77
Middle occipital gyrus	R	19	27	24	−91	2	3.90

**Children*: English* > *Chinese***							

–							

#### The Main Effect of Age

**FIGURE 3:**
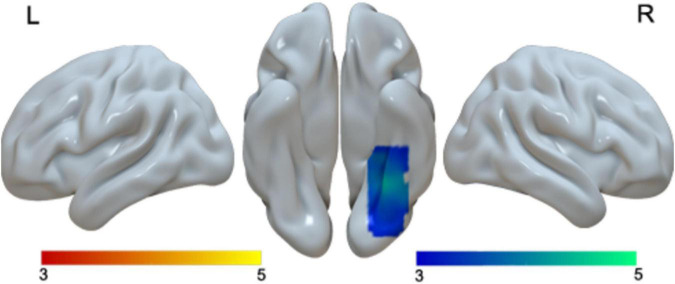
Brain activation in the main effect of age. Brain regions that were more activated in children than in adults (blue). No brain regions were more activated in adults than in children.

We found greater activation in children than in adults in the right fusiform gyrus (Table 2 and Figure 3). We found that no regions showed greater activation in adults than in children.

**FIGURE 4:**
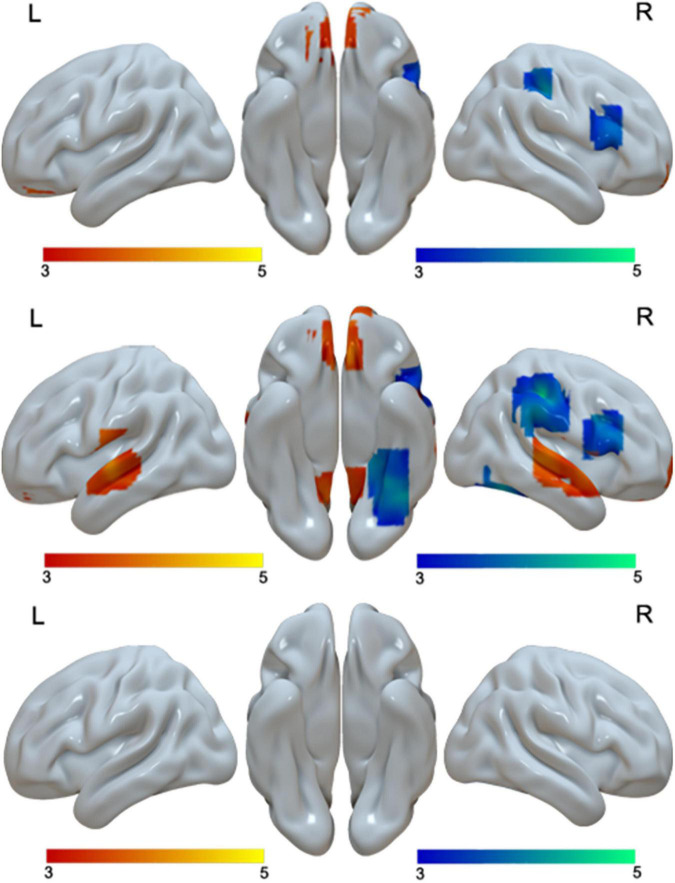
Brain activation in the interaction of language by age (first row), including brain activation in the positive interaction (red), and brain regions in the negative interaction (blue). Brain activation in the comparison between adults and children in English (second row). Brain regions that were more activated in adults than in children in English are in red; brain regions that were more activated in children than in adults are in blue. No brain regions showed age differences in Chinese (third row). The interactions were driven by greater age differences in English than in Chinese.

When we examined age effect separately in Chinese and English, we found no age differences in Chinese. However, for English, we found greater activation in adults than in children in bilateral medial frontal gyri, bilateral STG, and bilateral posterior cingulate gyri. We found greater activation in children than in adults in the right post-central gyrus, right IFG/MFG and the right fusiform gyrus for English (Table 4 and Figure 4).

**TABLE 4:** Brain regions that showed significant differences between adults and children in either English or Chinese.

Anatomical region	H	BA	Voxels	x	y	z	Z

English*: Adults* > *Children*							
Medial frontal gyrus	R/L	11	83	3	44	−13	4.38
Superior temporal gyrus	L	22	48	−57	−13	−1	4.32
Superior temporal gyrus	R	41,22	76	60	−25	5	4.12
Posterior cingulate gyrus	L/R	23	58	−6	−58	11	4.04

**English*: Children* > *Adults***							

Post-central gyrus, Inferior parietal lobule	R	40, 2	201	42	−34	44	5.02
Fusiform gyrus	R	37	80	33	−49	−16	4.74
Inferior frontal gyrus, Pre-central gyrus	R	6, 45	51	48	8	11	4.24

**Chinese: *Adults* > *children***							

–							

**Chinese: *Children* > *Adults***							

–							

#### Interaction Effect

At the whole brain level, we found three regions that showed a significant interaction effect between age and language (Table 2 and Figure 4). They are the right IFG/MFG, right post-central gyrus, and bilateral medial frontal gyrus. Break-down of the interaction showed that the interaction at the right IFG/MFG and the right post-central gyrus was due to greater activation in children than in adults in English but not Chinese, while the interaction at the bilateral medial frontal gyrus was due to greater activation in adults than in children in English but not in Chinese (Table 4 and Figure 4).

### Brain-Behavioral Correlation

**FIGURE 5:**
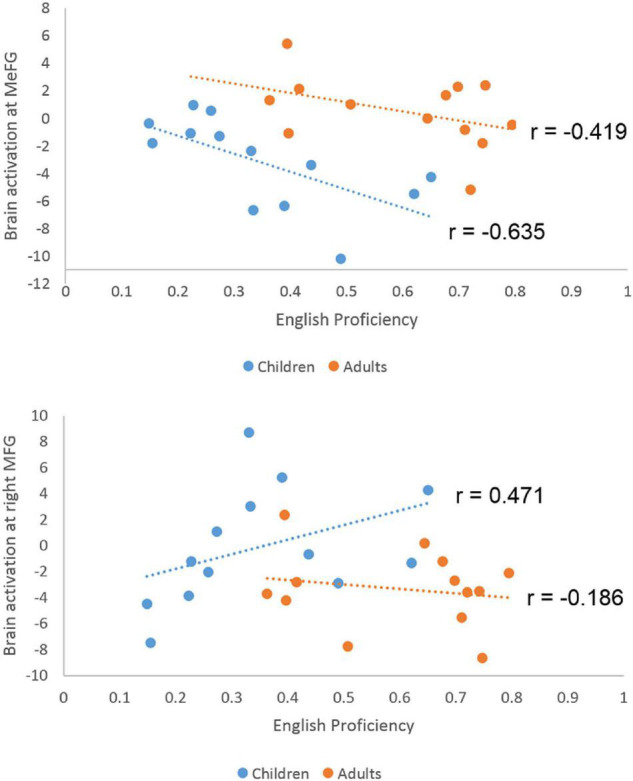
Scatter plots for the correlation between English proficiency and brain activation level at the medial frontal gyrus and right middle frontal gyrus in children and adults. Significant negative correlation was found in children but not in adults at the medial frontal gyrus. The correlation difference between children and adults is not significant. A marginally significant positive correlation was found in the right middle frontal gyrus for children.

Since the interactions were due to greater developmental differences in English than in Chinese, we correlated brain activation at the three regions that showed a significant interaction effect with English proficiency separately for children and adults. For the CE group, we calculated correlation between English proficiency and brain activation at the ROI of the right inferior frontal gyrus, post-central gyrus and the bilateral medial frontal gyrus. We found a significant negative correlation at the medial frontal gyrus (*r* = −0.635, *p* = 0.008) (Figure 5). We also found a marginally significant positive correlation at the right inferior frontal gyrus (*r* = 0.471, *p* = 0.066). The correlations in the AE group were not significant (*r* = −0.186, *p* = 0.543 for the right inferior frontal gyrus; *r* = 0.338, *p* = 0.259 for the right post-central gyrus; *r* = −0.419, *p* = 0.154 for the bilateral medial frontal gyrus). A direct comparison between the correlation coefficients in AE and CE at the medial frontal gyrus revealed a non-significant difference (*z* = −0.721, *p* = 0.47). It was not significantly different between AE and CE either at the right inferior frontal gyrus (*z* = 1.663, *p* = 0.09).

## Discussion

In this study, we examined the developmental changes in brain regions involved in L1 and L2 processing by directly comparing bilingual children and bilingual adults. Behaviorally, we found no interaction between age and language, which simply suggests that the proficiency level increased similarly in L1 and L2 from children to adults. However, brain imaging data revealed greater developmental changes in English than in Chinese. In terms of comparison between L1 and L2 within each age group, we found greater similarity between L1 and L2 in adults than in children, which supports the convergence hypothesis. When L2 proficiency increases, the L2 network and L1 network becomes more convergent.

### Language Differences in the Brain

For the main effect of language, we found greater activation in the left STG, IFG and bilateral lingual gyrus for Chinese than for English, and greater activation in the right superior parietal lobule for English than for Chinese. The differences at the left STG and IFG should be driven by different levels of semantic and phonological activation in Chinese and English. The stimuli for the English task were English pseudowords which elicit less semantic activation than Chinese words. The left STG is involved in phonological representation and language comprehension (Binder et al., 1997), while the left IFG is associated with phonological processing, such as phonological retrieval and phonological manipulation (Pugh et al., 1996; Fiez and Petersen, 1998). Greater activation in bilateral lingual gyri in Chinese than in English is due to more complex visuo-orthographic configuration in Chinese than in English, which is well documented in previous research (Bolger et al., 2005; Cao et al., 2010; Cao et al., 2013b). The greater activation in the right SPL in English than in Chinese might be due to compensation mechanisms of greater right hemispheric involvement in less proficient performers. The right SPL is associated with visuo-spatial processing as found in previous studies (Suchan et al., 2002). This region has been found to be more involved in Chinese than English (Cao et al., 2013b; Cao et al., 2015). Therefore, our finding suggests that these bilingual children and adults rely on visual spatial analysis important for their L1 to an even greater degree when they process L2, English, implicating a carry-over effect in L2 processing.

When we examined language difference in adults and children separately, we found greater language differences in bilingual children than bilingual adults. These additional language differences in children included greater activation in Chinese than in English at the right MTG and left caudate, as well as greater activation in English than in Chinese in the right SPL if we lowered the threshold. The DSC was 0.26 in adults and 0 in children. This is consistent with the convergence hypothesis that increased proficiency in L2 is associated with greater similarity to L1 brain activation (Perani et al., 1998; Golestani et al., 2006; Stein et al., 2009; Cao et al., 2013a). A previous study found that early bilinguals showed greater similarity between L1 and L2 at the left IFG than late bilinguals (Kim et al., 1997), suggesting an AOA effect. In our study, children had an earlier AOA than adults, however, we found greater similarity of brain activation in L1 and L2 in bilingual adults than bilingual children, suggesting a proficiency effect rather than an AOA effect. Our study adds to the literature that the overlap between L1 and L2 brain networks actually develops with learning and development. It provides important evidence from a developmental perspective for the convergence hypothesis, which argues that the difference between L1 and L2 in the brain disappears as L2 proficiency increases.

### Greater Developmental Changes in L2 Than in L1

The main effect of age was primarily driven by age differences in English, since we found no age differences in Chinese. For English, children had greater activation than adults in the right post-central gyrus, right inferior frontal gyrus and right fusiform gyrus, while adults had greater activation than children in bilateral medial frontal gyrus, bilateral STG and bilateral posterior cingulate gyrus. It suggests that child L2 learners who had a lower proficiency level tend to involve more right hemisphere regions. The involvement of the right hemisphere is common in beginning readers or low proficiency readers, such as those with dyslexia (Waldie et al., 2013). The involvement of the right IFG/MFG in younger normal children declines with age during reading (Shaywitz et al., 2007), suggesting that its importance decreases as reading develops in the left hemisphere. It has also been suggested that the right temporo-parietal-frontal regions are especially important for the early stage of language learning/acquisition in both L1 and L2 (Sugiura et al., 2011). Our finding is consistent with this pattern of shifting with learning. We found that the right IFG and post-central gyrus were more involved in bilingual children than bilingual adults for English, presumably due to compensation for deficient phonological processing in the left language network in children. Furthermore, at the right inferior frontal gyrus, there is a marginally significant positive correlation with English proficiency in children, suggesting that higher skilled children tend to use this right IFG compensation strategy to a greater degree. The right fusiform gyrus was also more involved in bilingual children than bilingual adults for English. This region is more involved in Chinese reading than in alphabetic reading (Bolger et al., 2005; Tan et al., 2005), presumably due to the more complex visuo-orthographic features of Chinese. It is also more involved in higher skilled Chinese readers than lower skilled readers (Cao et al., 2009; Cao et al., 2010), suggesting its essential role in Chinese reading. Therefore, greater activation in this region in children than in adults during English pseudoword processing suggests a greater reliance on the L1’s visuo-orthographic strategy when processing unfamiliar L2’s orthography.

On the other hand, adults showed greater activation than children in bilateral STG, bilateral medial frontal gyrus and bilateral posterior cingulate gyri during the English task. Greater activation in bilateral STG implicates greater activation level of English phonology which should be driven by greater familiarity to the English phonology in adults than in children. Bilateral medial frontal gyri and bilateral posterior cingulate gyri are critical parts of the default mode network (Raichle et al., 2001), which are deactivated during active cognitive tasks. Greater activation in these regions in adults than in children might be due to reduced task difficulty for adults than for children. We found a negative correlation with English proficiency in children in the bilateral medial frontal gyrus, suggesting that higher skilled children tend to deactivate this region to a greater degree.

### Limitations

One limitation of the current study is the cross-sectional between-subject design. It would be ideal to study developmental changes in the brain with language learning in a longitudinal design, however, due to obvious constraints, most studies conducted in the field of cognitive developmental neuroscience are cross-sectional. Even though it cannot provide strong causal inferences of brain development, the current study showed differences in the brain between adults and children during language processing, implicating age and proficiency influence in the brain. More importantly, the main finding of the study is the interaction between age and language; specifically, the age effect in L1 and L2 is different. Therefore, even if we employed a within-subject design, the key finding is still a between- subject comparison. Another limitation of the current study is the unmatched AOA in adults and children. If the AOA was matched, the proficiency effect would be even greater in the current study, because earlier AOA was associated with greater similarity between L1 and L2 (Kim et al., 1997), and adults had a later AOA but greater similarity than children in the current study. However, it is impossible to match AOA in Chinese bilingual children and adults due to national policy change. Future research should match AOA while examining age and proficiency effect.

## Conclusion

We examined the developmental change of the dynamics between L1 and L2 in the brain in Chinese-English late bilingual children and adults. We found greater similarity between L1 and L2 in adults than in children, supporting the convergence hypothesis, which argues that the difference between L1 and L2 disappears as L2 proficiency increases. Moreover, we found greater differences between children and adults in the L2’s brain network than in the L1’s brain network. Our study, for the first time, demonstrated that there might be different brain mechanisms underlying L1 and L2 development.

## Data Availability Statement

The raw data supporting the conclusions of this article will be made available by the authors upon request, without undue reservation.

## Ethics Statement

The studies involving human participants were reviewed and approved by Beijing Normal University and Michigan State University. Written informed assent and consent were obtained from participants and their parents, respectively.

## Author Contributions

FC: conceptualization, writing, and supervision. XY: data collection. YF, WC, MD-G, GS, and ZW: data analysis. YD: supervision of data collection. All authors contributed to the article and approved the submitted version.

## Conflict of Interest

The authors declare that the research was conducted in the absence of any commercial or financial relationships that could be construed as a potential conflict of interest.

## Publisher’s Note

All claims expressed in this article are solely those of the authors and do not necessarily represent those of their affiliated organizations, or those of the publisher, the editors and the reviewers. Any product that may be evaluated in this article, or claim that may be made by its manufacturer, is not guaranteed or endorsed by the publisher.

## Footnotes

1
http://www.fil.ion.ucl.ac.uk/spm

## References

[B1] Beijing Language and Culture University (1990). *Frequency for Modern Chinese Words* (Unpublished corpus). Beijing: Beijing Language and Culture University.

[B2] Berken J. A. Gracco V. L. Chen J. K. Watkins K. E. Baum S. Callahan M. (2015). Neural activation in speech production and reading aloud in native and non-native languages. *Neuroimage* 112 208–217. 10.1016/j.neuroimage.2015.03.016 25776210

[B3] Binder J. R. Frost J. A. Hammeke T. A. Cox R. W. Rao S. M. Prieto T. (1997). Human brain language areas identified in functional magnetic resonance imaging. *J. Neurosci.* 17 353–362. 10.1523/jneurosci.17-01-00353.1997 8987760 PMC6793702

[B4] Bolger D. J. Perfetti C. A. Schneider W. (2005). Cross-cultural effect on the brain revisited: universal structures plus writing system variation. *Hum. Brain Mapp.* 25 92–104. 10.1002/hbm.20124 15846818 PMC6871743

[B5] Briellmann R. S. Saling M. M. Connell A. B. Waites A. B. Abbott D. F. Jackson G. D. (2004). A high-field functional MRI study of quadri-lingual subjects. *Brain Lang.* 89 531–542. 10.1016/j.bandl.2004.01.008 15120544

[B6] Cao F. Brennan C. Booth J. R. (2015). The brain adapts to orthography with experience: evidence from English and Chinese. *Dev. Sci.* 18 785–798. 10.1111/desc.12245 25444089 PMC4934123

[B7] Cao F. Lee R. Shu H. Yang Y. Xu G. Li K. (2010). Cultural constraints on brain development: evidence from a developmental study of visual word processing in Mandarin Chinese. *Cereb. Cortex* 20 1223–1233. 10.1093/cercor/bhp186 19773547 PMC2852508

[B8] Cao F. Peng D. Liu L. Jin Z. Fan N. Deng Y. (2009). Developmental differences of neurocognitive networks for phonological and semantic processing in Chinese word reading. *Hum. Brain Mapp.* 30 797–809. 10.1002/hbm.20546 18330872 PMC2823253

[B9] Cao F. Tao R. Liu L. Perfetti C. A. Booth J. R. (2013a). High proficiency in a second language is characterized by greater involvement of the first language network: evidence from Chinese learners of English. *J. Cogn. Neurosci.* 25 1649–1663. 10.1162/jocn_a_00414 23654223 PMC3979436

[B10] Cao F. Vu M. Chan D. H. Lawrence J. M. Harris L. N. Guan Q. (2013b). Writing affects the brain network of reading in Chinese: a functional magnetic resonance imaging study. *Hum. Brain Mapp.* 34 1670–1684. 10.1002/hbm.22017 22378588 PMC6870511

[B11] Chee M. W. Caplan D. Soon C. S. Sriram N. Tan E. W. Thiel T. (1999a). Processing of visually presented sentences in Mandarin and English studied with fMRI. *Neuron* 23 127–137. 10.1016/s0896-6273(00)80759-x 10402199

[B12] Chee M. W. Tan E. W. Thiel T. (1999b). Mandarin and English single word processing studied with functional magnetic resonance imaging. *J. Neurosci.* 19 3050–3056. 10.1523/JNEUROSCI.19-08-03050.1999 10191322 PMC6782281

[B13] Chee M. W. Weekes B. Lee K. M. Soon C. S. Schreiber A. Hoon J. J. (2000). Overlap and dissociation of semantic processing of Chinese characters, English words, and pictures: evidence from fMRI. *Neuroimage* 12 392–403. 10.1006/nimg.2000.0631 10988033

[B14] Cherodath S. Singh N. C. (2015). The influence of orthographic depth on reading networks in simultaneous biliterate children. *Brain Lang.* 143 42–51. 10.1016/j.bandl.2015.02.001 25747965

[B15] Costa A. Pannunzi M. Deco G. Pickering M. J. (2016). Do bilinguals automatically activate their native language when they are not using it? *Cogn. Sci.* 41 1629–1644. 10.1111/cogs.12434 27766658

[B16] Craddock R. C. James G. A. Holtzheimer P. E. III Hu X. P. Mayberg H. S. (2012). A whole brain fMRI atlas generated via spatially constrained spectral clustering. *Hum. Brain Mapp.* 33 1914–1928. 10.1002/hbm.21333 21769991 PMC3838923

[B17] De Bleser R. Dupont P. Postler J. Bormans G. Speelman D. Mortelmans L. (2003). The organisation of the bilingual lexicon: a PET study. *J. Neurolinguistics* 16 439–456. 10.1007/s11910-002-0037-y 12359104

[B18] Dice L. R. (1945). Measures of the amount of ecologic association between species. *Ecology* 26 297–302.

[B19] Fiez J. A. Petersen S. E. (1998). Neuroimaging studies of word reading. *Proc. Natl. Acad. Sci. U.S.A* 95 914–921. 10.1073/pnas.95.3.914 9448259 PMC33816

[B20] Golestani N. Alario F. X. Meriaux S. Le Bihan D. Dehaene S. Pallier C. (2006). Syntax production in bilinguals. *Neuropsychologia* 44 1029–1040. 10.1016/j.neuropsychologia.2005.11.009 16427099

[B21] Green D. W. (2003). “The neural basis of the lexicon and the grammar in L2 acquisition,” in *The Interface Between Syntax and the Lexicon in Second Language Acquisition*, eds van Hout A. H. R. Kuiken F. Towell R. (Amsterdam: John Benjamins), 197–208. 10.1080/02643294.2014.882814

[B22] Hernandez A. E. Hofmann J. Kotz S. A. (2007). Age of acquisition modulates neural activity for both regular and irregular syntactic functions. *Neuroimage* 36 912–923. 10.1016/j.neuroimage.2007.02.055 17490895 PMC1995424

[B23] Hernandez A. E. Woods E. A. Bradley K. A. (2015). Neural correlates of single word reading in bilingual children and adults. *Brain Lang.* 143C 11–19. 10.1016/j.bandl.2015.01.010 25728012 PMC5944362

[B24] Ip K. I. Hsu L. S. Arredondo M. M. Tardif T. Kovelman I. (2016). Brain bases of morphological processing in Chinese-English bilingual children. *Dev. Sci.* 20 12449. 10.1111/desc.12449 PMC5309206 27523024

[B25] Johnson M. H. (2011). Interactive specialization: a domain-general framework for human functional brain development? *Dev. Cogn. Neurosci.* 1 7–21. 10.1016/j.dcn.2010.07.003 22436416 PMC6987575

[B26] Kim K. H. S. Relkin N. R. Lee K. M. Hirsch J. (1997). Distinct cortical areas associated with native and second languages. *Nature* 388 171–174. 10.1038/40623 9217156

[B27] Klein D. Milner B. Zatorre R. J. Zhao V. Nikelski J. (1999). Cerebral organization in bilinguals: a PET study of Chinese-English verb generation. *NeuroReport* 10 2841–2846. 10.1097/00001756-199909090-00026 10511450

[B28] Koda K. (1990). Factors affecting second language text comprehension. *Natl. Read. Conference Yearb.* 39 419–427.

[B29] Kroll J. F. Bogulski C. A. McClain R. (2012). “Psycholinguistic perspectives on second language learning and bilingualism: the course and consequence of cross-language competition,” in *Linguistic Approaches to Bilingualism* (Amsterdam: John Benjamnins), 1–24. 10.1075/lab.2.1.01kro

[B30] MacWhinney B. (2012). “The logic of the unified model,” in *The Routledge Handbook of Second Language Acquisition*, eds Gass S. M. Mackey A. (Abingdon: Routledge), 17.

[B31] Musso M. Moro A. Glauche V. Rijntjes M. Reichenbach J. Buchel C. (2003). Broca’s area and the language instinct. *Nat. Neurosci.* 6 774–781. 10.1038/nn1077 12819784

[B32] Perani D. Abutalebi J. (2005). The neural basis of first and second language processing. *Curr. Opin. Neurobiol.* 15 202–206. 10.1016/j.conb.2005.03.007 15831403

[B33] Perani D. Paulesu E. Galles N. S. Dupoux E. Dehaene S. Bettinardi V. (1998). The bilingual brain: proficiency and age of acquisition of the second language. *Brain* 121 1841–1852. 10.1093/brain/121.10.1841 9798741

[B34] Pugh K. R. Shaywitz B. A. Shaywitz S. E. Constable R. T. Skudlarski P. Fulbright R. K. (1996). Cerebral organization of component processes in reading. *Brain* 119 1221–1238. 10.1093/brain/119.4.1221 8813285

[B35] Raichle M. E. MacLeod A. M. Snyder A. Z. Powers W. J. Gusnard D. A. Shulman G. L. (2001). A default mode of brain function. *Proc. Natl. Acad. Sci. U.S.A* 98 676–682. 11209064 10.1073/pnas.98.2.676 PMC14647

[B36] Sakai K. L. Miura K. Narafu N. Muraishi Y. (2004). Correlated functional changes of the prefrontal cortex in twins induced by classroom education of second language. *Cereb. Cortex* 14 1233–1239. 10.1093/cercor/bhh084 15142962

[B37] Shaywitz B. A. Skudlarski P. Holahan J. M. Marchione K. E. Constable R. T. Fulbright R. K. (2007). Age-related changes in reading systems of dyslexic children. *Ann. Neurol.* 61 363–370. 10.1002/ana.21093 17444510

[B38] Stein M. Federspiel A. Koenig T. Wirth M. Lehmann C. Wiest R. (2009). Reduced frontal activation with increasing 2nd language proficiency. *Neuropsychologia* 47 2712–2720. 10.1016/j.neuropsychologia.2009.05.023 19501603

[B39] Suchan B. Yaguez L. Wunderlich G. Canavan A. G. Herzog H. Tellmann L. (2002). Neural correlates of visuospatial imagery. *Behav. Brain Res.* 131 163–168. 10.1016/s0166-4328(01)00373-4 11844583

[B40] Sugiura L. Ojima S. Matsuba-Kurita H. I Tsuzuki D. Katura T. Hagiwara H. (2011). Sound to language: different cortical processing for first and second languages in elementary school children as revealed by a large-scale study using fNIRS. *Cereb. Cortex* 21 2374–2393. 10.1093/cercor/bhr023 21350046 PMC3169662

[B41] Tan L. H. Laird A. R. Karl L. Fox P. T. (2005). Neuroanatomical correlates of phonological processing of Chinese characters and alphabetic words: a meta-analysis. *Hum. Brain Mapp.* 25 83–91. 10.1002/hbm.20134 15846817 PMC6871734

[B42] Tettamanti M. Alkadhi H. Moro A. Perani D. Kollias S. Weniger D. (2002). Neural correlates for the acquisition of natural language syntax. *Neuroimage* 17 700–709. 10.1006/nimg.2002.1201 12377145

[B43] Waldie K. E. Haigh C. E. Badzakova-Trajkov G. Buckley J. Kirk I. J. (2013). Reading the wrong way with the right hemisphere. *Brain Sci.* 3 1060–1075. 10.3390/brainsci3031060 24961521 PMC4061874

[B44] Wartenburger I. Heekeren H. R. Abutalebi J. Cappa S. F. Villringer A. Perani D. (2003). Early setting of grammatical processing in the bilingual brain. *Neuron* 37 159–170. 10.1016/s0896-6273(02)01150-9 12526781

[B45] Wu Y. J. Thierry G. (2010). Chinese-English bilinguals reading English hear Chinese. *J Neurosci* 30 7646–7651. 10.1523/JNEUROSCI.1602-10.2010 20519539 PMC6632379

[B46] Woodcock R. W. McGrew K. S. Mather N. (2001). *Woodcock-Johnson III Tests of Achievement.* Itasca, IL: The Riverside Publishing Company.

[B47] Xue G. Dong Q. Jin Z. Zhang L. Wang Y. (2004). An fMRI study with semantic access in low proficiency second language learners. *Neuroreport* 15 791–796. 10.1097/00001756-200404090-00010 15073516

